# Fluid bolus increases plasma hyaluronan concentration compared to follow-up strategy without a bolus in oliguric intensive care unit patients

**DOI:** 10.1038/s41598-024-71670-2

**Published:** 2024-09-06

**Authors:** Maija Serlo, Nina Inkinen, Päivi Lakkisto, Miia Valkonen, Anni Pulkkinen, Tuomas Selander, Ville Pettilä, Suvi T. Vaara

**Affiliations:** 1 Department of Perioperative and Intensive Care, Intensive and Intermediate Care Unit, University of Helsinki and Helsinki University Hospital, P.O. Box 320, 00290 HUS Helsinki, Finland; 2 Department of Anesthesia and Intensive Care, Hospital Nova of Central Finland, Central Finland Health Care District, Hoitajantie 3, 40620 Jyväskylä, Finland; 3 Department of Clinical Chemistry and Hematology, University of Helsinki and Helsinki University Hospital, Helsinki, Finland; 4 Minerva Foundation Institute for Medical Research, Helsinki, Finland; 5 Science Service Center, Kuopio University Hospital, Kuopio, Finland

**Keywords:** Angiopoietins, Endothelium, Endothelial glycocalyx, Fluid therapy, Hyaluronan, Tie1, Biomarkers, Clinical trial design, Biomarkers

## Abstract

Fluid therapy is a fundamental part of supportive therapy in critical care. However, it is also a suspected risk for endothelial glycocalyx degradation which is associated with poor clinical outcomes. This secondary analysis of RESPONSE randomized trial compares the effect of follow-up strategy (FU) on endothelial biomarkers to that of 500 ml crystalloid fluid bolus (FB) in oliguric, hemodynamically optimized intensive care unit (ICU) patients. 130 adult subjects were enrolled in two Finnish ICUs from January 2017 to November 2020. Blood and urine samples of 63 patients in FU group and 67 patients in FB group were collected before and after the intervention and analyzed using enzyme-linked immunosorbent assays. Single fluid bolus, given after median of 3887 ml (interquartile range 2842; 5359 ml) resuscitation fluids in the preceding 24 h, increased plasma hyaluronan concentration compared to the follow-up strategy (difference in medians 29.2 ng/ml with 95% CI [14.5ng/ml; 55.5ng/ml], *P* < 0.001). No treatment effect was detected in the plasma levels of syndecan-1, , angiopoietin-2, angiopoietin receptors Tie2 and Tie1, or in soluble thrombomodulin in the adjusted median regression analysis. The increase in hyaluronan was independent of its simultaneous renal clearance but correlated moderately with the increase in endothelium-specific Tie1. The follow-up strategy did not show consistent endothelium-sparing effect but protected against hyaluronan increase. The mechanisms and consequences of hyaluronan fluctuations need further clarification. Trial registration: clinicaltrials.gov, NCT02860572. Registered 1 August 2016, https://www.clinicaltrials.gov/study/NCT02860572?term=NCT02860572&rank=1

## Introduction

Endothelial dysfunction and subsequent microcirculatory failure are common denominators of organ dysfunction in critical illness^1^. Soluble molecules of endothelium and its glycocalyx (EG), a luminal regulatory layer of the endothelium, have been used as indicators of EG degradation and endothelial dysfunction^1^. Altered plasma levels of these molecular biomarkers have been associated with outcomes in many clinical entities, e.g. sepsis, acute kidney injury (AKI), adult respiratory distress syndrome and trauma^2–7^.

The general goal of fluid therapy is to increase cardiac output to secure adequate tissue perfusion^8^. However, some studies suggest that large volume or rapid administration of fluid could harm the structure of the EG which actually could impair the microcirculation^9–11^. Yet, others have not found such an association^12,13^. Supportive evidence for fluid-induced effects on endothelium comes mainly from studies concerning healthy volunteers or stable surgical patients^14^. Results from critically ill cohorts are more conflicting and prone to many factors that interfere with the detection and interpretation of alterations in endothelium-related biomarkers^14^. AKI and oliguria have been claimed as potent confounders^15^. In addition, it is not known whether previous fluid therapy or already manifested endothelial injury alters endothelial responses to additional fluid-therapy.

The basic structure of the EG consists of a network of proteoglycans and glycoproteins to which numerous other molecules and serum proteins connect^16^. The proteoglycans comprise of glycosaminoglycan-sidechains attached to membrane-bound core proteins, e.g., syndecan-1 (SDC-1)^16^. Heparan sulfate is the most prominent glycosaminoglycan that interconnects specific regulatory molecules to the EG via electrostatic interactions^16^. Another glycosaminoglycan, hyaluronan (HA), affects vessel permeability, mechanosensory effect and contributes to the regulation of inflammation and thrombosis^17^. The angiopoietin-Tie system is a key vascular regulatory system that may serve as a link between inflammation, fluid therapy, and EG disruption^6,18–21^. This system comprises vascular stability promoting Tie2 and modulatory Tie1 tyrosine-protein kinase receptors and three angiopoietin ligands, ANG-1, ANG-2 and ANG-4. ANG-1 and ANG-4 are pure agonists, but ANG-2 serves context-dependently either as an agonist or an antagonist^18,22^. Tie2 antagonism leads to endothelial activation that manifests as increased expression of adhesion molecules for leukocytes, weakened intercellular junctions and increased amount of pro-coagulant proteins at the luminal surface^18^. High plasma levels of ANG-2 have constantly been shown to associate with increased vascular permeability and to reflect severity of illness in critically ill patients^18,20,23^. Liberal fluid resuscitation has been associated to higher ANG-2 levels over time compared to lower levels with restrictive dosing^6^. Mechanistic links between the components of EG and angiopoietin-system have been proposed^19–21^. Tie1 is little studied in critical care context.

Randomized, controlled trial setups are highly needed to unravel the aforementioned uncertainties of the effects of fluid therapy on the endothelium in the critically ill. Accordingly, we conducted this secondary analysis on our previous randomized clinical trial^24^ to explore the hypothesis that fluid-therapy approach at a single bolus level affects the biomarkers of EG and members in the ANG-Tie-signaling cascade independently of renal clearance in critically ill patients after initial fluid resuscitation.

### Objectives

The primary objective of this secondary analysis of RESPONSE trial was to compare the effect of non-interventional follow-up on endothelial biomarkers to that of a fluid bolus in oliguric ICU patients. Second, we assessed whether the potential changes were attributable to simultaneous changes in marker-specific renal clearances and, whether the changes associated with the baseline-level of endothelial dysfunction marker ANG2. Third, we scrutinized whether the changes in the studied functional and structural entities associated with each other.

## Methods

The RESPONSE trial (clinicaltrials.gov NCT02860572) was a two-center randomized, controlled, open-label pilot trial conducted in two Finnish ICUs. The study protocol was approved by the Ethics Committee of the Department of Surgery, Hospital District of Helsinki and Uusimaa (Decision number HUS/1308/2016). The protocol and results of the main trial have been published^24^. The trial was conducted according to the Declaration of Helsinki and its later amendments and according to Good Clinical Practice guidelines.

### The study design and participants

This trial includes all patients originally enrolled in the RESPONSE trial. Oliguric (urine output < 0,5ml/kg/h at least for 2 h), acutely admitted adult ICU-patients were randomized to either to the FU (no boluses, no diuretics) and FB (500ml of Ringer’s acetate solution over 30 min intravenously) groups^24,25^. Briefly, the patients had received initial resuscitation and were hemodynamically stabilized before study inclusion. Ongoing infusions and nutrition were kept constant in both groups throughout the study period without protocol demanding exact infusion rates. Mean arterial pressure targets were not modified during the study period, but vasoactive drugs, sedation, insulin and other drug infusions were titrated according to the treating clinician. Rescue bolus of 500 ml crystalloid was allowed in both groups if predefined criteria for suspected hypovolemia were fulfilled. The RESPONSE trial showed that FU-strategy improved the urine output less frequently but led to lower positive cumulative fluid balance compared to FB.

Patients were randomized in 1:1 ratio to both FB and FU groups. Randomization was stratified with the presence/absence of sepsis according to sepsis-3 definition^26^ and AKI according to KDIGO-criteria^27^ using permuted blocks of varying size (4, 6 and 8). Each patient or their next of kin gave written informed consent for the study, primarily before trial enrollment whenever possible. If not possible, deferred consent was used due to the time-sensitive nature of the intervention and the critical illness of the patients. Patients were recruited from January 2017 to November 2020.

The full list of inclusion and exclusion criteria is presented in Supplemental Table S1. In short, we screened all adult (18 years or older), not pregnant or lactating, emergently admitted patients without chronic kidney disease (CKD) (estimated GFR > 60ml/min/1,72m2) and without urgent need for renal replacement therapy for initial eligibility. Of those, we enrolled the patients without exclusion criteria, who developed oliguria lasting at least two consecutive hours within 72 h from the admission to the ICU. We excluded patients with (1) marked fluctuations in hemodynamics, (2) bleeding or (3) ongoing initial fluid resuscitation, (4) no consent, (5) expected survival less than 24 h, (6) fluid overload, pulmonary edema or increased intra-abdominal pressure and (7) those who had received furosemide within the last 6 h. The flow diagram of all screened patients has been published^24^.

### Biomarkers

We measured a set of mechanistically and structurally linked biomarkers from plasma and urine sampled before and after the intervention period. The biomarkers were SDC-1, and HA reflecting EG injury; soluble thrombomodulin (sTM), a conventional marker of endothelial cell injury, and ANG-2 indicating endothelial dysregulation. Additionally, we measured soluble domains of Tie2 and of Tie1 from plasma.

### Blood and urine sampling

**Fig. 1:**
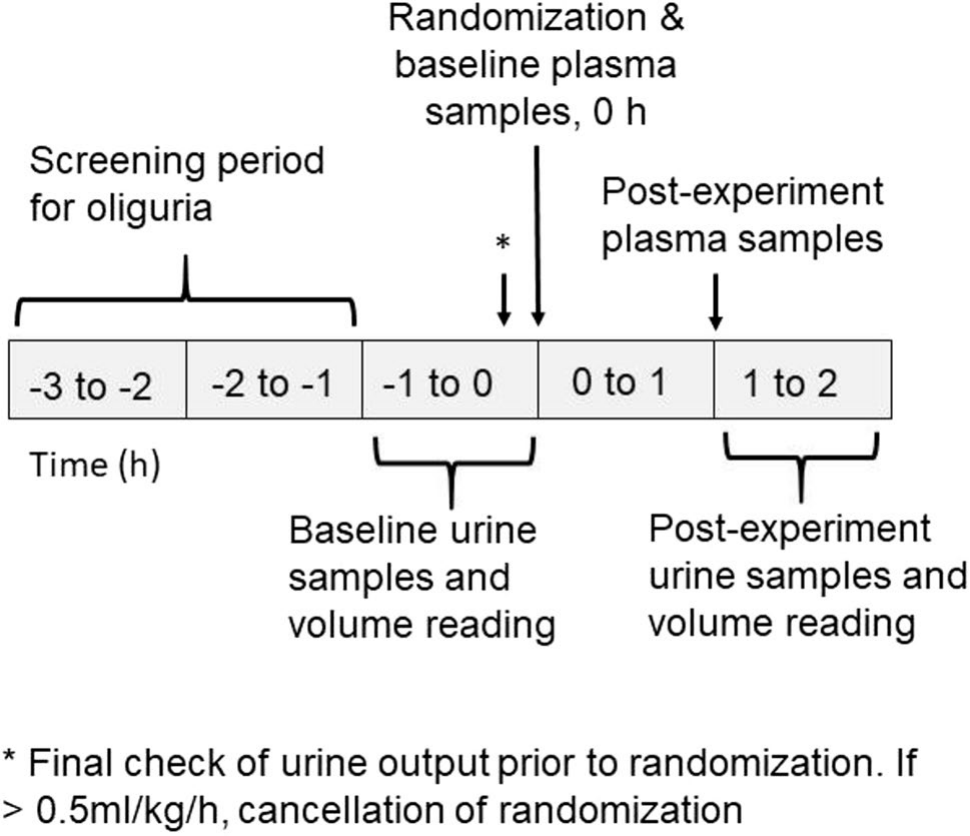
Timeline of the experiment.

Plasma samples were collected immediately before the beginning of the intervention period and subsequently 60 min later. These time-points were determined based on scarce data about the kinetics of plasma HA, SDC-1 and ANG-2 in healthy subjects after comparable fluid boluses^9,28,29^. Blood samples were drawn through an arterial catheter to heparin tubes. Blood samples were centrifuged at 2000 g for 10 min, aliquoted and stored at − 70 °C before assays. Available urine samples represent the urine passed during the previous hour before sampling. First urine sample was collected immediately before the beginning of the intervention period. Second sample was collected 2 h after the intervention. Urine samples were taken via a standard urinometer. Urine volume was recorded hourly. Urine samples were centrifuged at 400g for 10 min and stored at − 70 °C. Figure 1 shows the timeline of the experiment.

### Enzyme-linked immunosorbent assays and other biochemical measurements

We performed ELISAs using commercially available kits for SDC-1 (Diaclone SAS, Besançon, France), for HA, sTM, ANG-2 and for Tie2 (Quantikine™ ELISA, R&D systems, Minneapolis, USA) according to the respective protocols provided by the manufacturers. Tie1 was measured using Human Tie1 DuoSet® ELISA development system and DuoSet® Ancillary Reagent Kit 2 (R&D Systems, Minneapolis, USA) according to manufacturer’s instructions. Intra- and inter-assay coefficients of variation, CV (%) were calculated for each assay. Please see the supplemental Methods for a more detailed description. The urine albumin-to-creatinine ratio (ACR) was analyzed in an accredited clinical laboratory using standard photometric immunochemical method for albumin and photometric enzymatic method for creatinine. Hemoglobin values were analyzed as a part of the bedside arterial blood gas analyses in calibrated automatized blood-gas-analyzer.

### Missing values

Those samples that fell below the detection limit of a given ELISA-assay were assigned as mean minimal detectable dose, given by the manufacturer. Upper detection limit values were imputed for samples exceeding the upper detection limit after repeated dilutions. Those samples that behaved non-linearly between different dilutions, or when reanalysis in different dilution would have been necessary but not possible due to technical issues, were considered erroneous and thus, as missing.

### Calculations for the estimates of renal clearances

We calculated estimates of renal clearances of solutes X (HA, SDC-1, sTM and ANG-2) at the baseline (BL) and after the study period (post) based on our available plasma and urine data. We used the formulas Clearance (X)_BL_ = U(X)_BL_ * V_BL_/P(X)_BL_ and Clearance (X)_post_ = U(X)_post_ * V_post_/P(X)_post_. U(X)_BL_ and U(X)_post_ indicate the urine concentration of solute X in the representative time points (please see Fig. 1), V_BL_ and V_post_ indicate the urine volume per the respective 60-min collection periods and P(X)_BL_ and P(X)_post_ indicate the plasma concentrations of solute X in the respective time-points.

### Statistical analysis

The study was powered according to the primary endpoint in the parent study. Thus, no formal power calculations were made. We performed statistical analyses on the modified intention-to-treat population and additional sensitivity analyses on the per-protocol population as described previously^24,25^. We conducted subgroup analysis for plasma biomarkers in septic and AKI-patient-groups. To control any remaining hemodilution effects, we conducted an additional sensitivity analysis for the plasma biomarkers adjusted to the simultaneously sampled hemoglobin values.

We tested continuous variables for normality using the Shapiro–Wilk test. We report categorical variables as counts with percentages and non-normally distributed, continuous variables as medians and interquartile ranges (IQR). We compared independent groups with chi-square or Mann–Whitney U tests. We compared the effect of the fluid-therapy strategy on plasma and urine biomarkers using median regression adjusting with the stratification factors and the baseline levels of each biomarker. We report results of regression analyses as difference in medians with 95% confidence intervals (CIs). We test whether the biomarker-specific renal clearances differ within groups over the study period using Wilcoxon Matched-Pair Signed-Rank test and report medians of differences (post–pre) with IQRs. We analyzed the associations between biomarkers using Spearman’s rho nonparametric correlation. Due to the explorative nature of our study, we did not adjust the significance level for multiple comparisons^30^. We used R statistical software version 3.6.2 and SPSS Statistics 27.

## Results

Overall, 130 patients were randomized, 67 patients to FB group and 63 to the FU group. Plasma samples were available from all patients. Table 1 shows the baseline clinical characteristics of the patients and Table 2 the baseline biomarker concentrations. FB group received total of 625 ml (586 ml; 684 ml) of fluids during the intervention hour and FU group 120 ml (98 ml; 168 ml), *P* < 0,001. One patient in FU group received a rescue fluid bolus.

**Table 1:** Baseline patient characteristics.

Characteristic	Non-interventional follow-up, *N* = 63	Fluid bolus, *N* = 67
Age (years)	67 (53—72)	69 (59—76)
Sex; male (%)	44 (69.8)	41 (61.2)
Weight (kg)	85 (75—102)	83 (73—100)
SAPS II score	37 (34—45)	41 (33—48)
Surgical admission n (%)	33 (52.4)	37 (55.2)
Time from the admission to ICU, h	21 (13—35)	19 (11—29)
AKI, (%)	37 (58.7)	40 (59.7)
Sepsis, (%)	35 (55.6)	37 (55.2)
Previous 24h intake of fluids (ml)	4381 (2921; 5918)	3887 (2842; 5359)
Cumulative balance of fluids at ICU before randomization (ml)	1856 (1296—2948)	1941 (1201—3762)
SOFA score	7 (6—10)	7 (5—9)
Missing urine samples n (%)	11 (17.5)	11 (16.4)

Data included from all 130 patients. Data are *n* (%) or median (interquartile range). SAPS II: Simplified Acute Physiology Score II, ICU Intensive Care Unit, AKI Acute Kidney Injury according to KDIGO criteria, SOFA Sequential Organ Failure Assessment; all six organ systems considered. Missing urine samples refer to patients in whom scarcity of urine prevented sampling.

**Table 2:** Baseline Biomarker concentrations.

Biomarker	Non-interventional follow-up, *N* = 63	Fluid bolus, *N* = 67
Plasma biomarkers for glycocalyx disruption (ng/ml)		
SDC-1	133 (87.5; 265)	138 (71.4; 259)
HA	117 (54.0; 260)	112 (49.4; 327)
Plasma biomarker of endothelial cell injury (ng/ml)		
sTM	6.50 (4.04; 9.95)	6.12 (4.36; 11.57)
Plasma biomarkers for endothelial dysregulation/activation (ng/ml)		
ANG-2	7.40 (4.67; 12.70)	7.18 (4.42; 14.05)
Tie2	14.97 (11.19; 19.81)	14.84 (11.25; 19.51)
Tie1	21.42 (16.82; 35.12)	21.31 (15.35; 39.49)
Urine biomarkers (ng/ml)		
U-SDC-1	282 (164; 564)	275 (172; 400)
U-HA	95 (72; 128)	105 (86; 144)
U-sTM	25.5 (13.0; 36.5)	20.2 (12.1; 29.6)
U-ANG-2	0.23 (0.07; 0.85)	0.23 (0.03; 0.88)
ACR (mg/mmol)	5.55 (2.05; 12,3)	5.06 (2,77; 14.6)
Urine/plasma ratio of SDC-1	2.13 (1.09; 4.03)	2.22 (1.14; 3.69)
Urine/Plasma ratio of HA	0.83 (0.53; 1.86)	1.03 (0.46; 2.10)
Urine/plasma ratio of sTM	3.85 (1.86; 7.76)	2.82 (1.51; 5.54)
Urine/Plasma ratio of ang2	0.02 (0.00; 0.11)	0.03 (0.01; 0.13)

SDC-1: syndecan-1, HA: hyaluronan, sTM: soluble thrombomodulin, ANG-2: angiopoietin 2, Tie2 and Tie1: Tyrosine-protein kinase receptors Tie 2 and 1, ACR: albumin to creatinine ratio in urine.

Baseline data of 22 (16.9%) patients, whose urine volume in one or both sampling points was too low for urine ELISA-analyses are represented in the Supplemental Table S2. Those patients were more severely ill as reflected by higher baseline Simplified Acute Physiology Score II (SAPS II) and Sequential Organ Failure Assessment (SOFA) scores, had higher plasma sTM and Tie1 levels, but were evenly distributed between FB and FU groups (11:11). Supplemental Table S3 presents CVs for the ELISAs. The assays showed robust performance.

### Plasma biomarkers

**Fig. 2:**
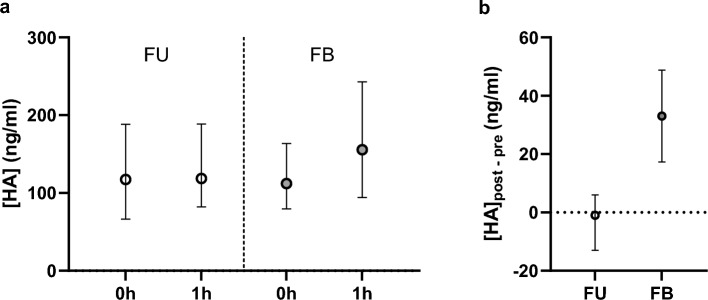
Hyaluronan concentrations in plasma. (**a**) Hyaluronan concentration in follow up (FU) and in fluid bolus (FB) groups before (0h) and after (1h) the intervention (median with 95% CI). (**b**) Individual changes in the hyaluronan concentration during the intervention (median with 95% CI).

The plasma concentration of HA was significantly higher in the FB group than in the FU group after the intervention (difference in medians 29.2 ng/ml with 95% CI [14.5 ng/ml; 55.5 ng/ml] *p* < 0,001), while no differences were seen in any other of the studied biomarkers in the adjusted median regression analysis (Table 3, Fig. 2). The results were comparable in unadjusted analysis (Supplemental Table S4) and when biomarker concentrations were normalized to concomitantly measured hemoglobin (Supplemental Table S5). Fluid bolus increased the plasma HA concentration also in the subgroup of septic patients (difference in medians 56.9ng/ml, [28.9 ng/ml; 109 ng/ml], *P* < 0.001) (Supplemental Table S6) and in AKI-patients (difference in medians 20.5 ng/ml, [4.29; 45.5], *P* = 0.009) (Supplemental Table S7). Results were similar in per-protocol population (Supplemental Table S8). A small increase after the fluid bolus was seen in SDC-1/Hb ratio in FB group, but the difference between groups was not significant (Supplemental Table S5).

**Table 3:** Biomarkers in plasma at 1 h.

Biomarker (ng/ml)	Non-interventional follow-up	Fluid bolus	Difference in medians [95% CI]	*P*^a^
SDC-1	133 (80.2; 280)	140 (81.1; 256)	4.94 [− 5.11; 19.8]	0.245
HA	119 (43.0; 324)	156 (69.0; 418)	29.2 [14.5; 55.5]	< 0.001
sTM	5.98 (4.39; 9.57)	7.00 (4.23; 10.46)	− 0.21 [-0.86; 0.40]	0.450
ANG-2	7.47 (4.54; 12.61)	7.44 (4.32; 13.81)	0.06 [− 0.36; 0.65]	0.752
Tie2	15.03 (12.44; 20.25)	14.09 (10.92; 19.48)	0.24 [− 1.44; 1.73]	0.824
Tie1	23.42 (18.28; 32.60)	23.06 (16.86; 35.46)	0.05 [− 3.87; 2.59]	0.732

Median regression analysis, ^a^adjusted to sepsis and AKI and the baseline values of each marker. Values are median (interquartile range) if not otherwise stated. Three plasma HA-concentrations in FU-group and two in FB-group fell above the upper detection limit and were assigned the limit value.

SDC-1: syndecan-1, HA: hyaluronan, sTM: soluble thrombomodulin, ANG-2: angiopoietin 2, Tie2 and Tie1: Tyrosine-protein kinase receptors Tie 2 and 1.

### Renal clearance and urinary data

The calculated renal clearances of all the studied biomarkers were minimal < 2ml/min and stayed stable in the FU-group (Table 4). HA clearance stayed stable also in the FB group (*P* = 0.958). Fluid bolus increased renal clearances of SDC-1, sTM and ANG-2 statistically significantly, although the absolute median clearances remained less than 2ml/min. Urinary concentrations of each biomarker after the study interventions and the urine-to-plasma concentration ratios are shown in Table 5. The urine albumin-to-creatinine ratios were comparable in both groups.

**Table 4:** Estimates of biomarker-specific renal clearances.

Clearance (ml/min)	Baseline	Post	Difference*	*P*
SDC-1				
FU	0.89 (0.35; 1.57)	1.23 (0.39; 1.59)	0.02 (− 0.40; 0.86)	0.276
FB	0.74 (0.39; 1,56)	1.22 (0.49; 1.99)	0.24 (− 0.10; 0.65)	0.002
HA				
FU	0.33 (0.08; 0.81)	0.38 (0.10; 0.88)	0.01 (− 0.27; 0.47)	0.436
FB	0.35 (0.11; 0.85)	0.41 (0.09; 0.74)	0.01 (− 0.25; 0.28)	0.958
sTM				
FU	1.50 (0.61; 3.14)	1.91 (0.57; 3.75)	0.20 (− 1.16; 1.23)	0.567
FB	0.94 (0.45; 2.68)	1.63 (0.57; 3.77)	0.37 (− 0.25; 1.41)	0.007
ANG-2				
FU	0.01 (0.00; 0.04)	0.01 (0.00; 0.03)	0.00 (− 0.01; 0.01)	0.210
FB	0.01 (0.00; 0.05)	0.02 (0.00; 0.07)	0.00 (− 0.00; 0.03)	0.002

Values are median (interquartile range). *Post minus pre values. SDC-1: syndecan-1, HA: hyaluronan, sTM: soluble thrombomodulin, ANG-2: angiopoietin 2.

**Table 5:** Urine biomarkers and urine/plasma ratios after the intervention.

Marker	FU	FB	Difference in medians [95% CI]	*p*^a^
ACR (mg/mmol)	5.54 (2.03; 14,1)	5.05 (2.05; 15,0)	− 0.68 [− 1.28; 0.59]	0.310
U-SDC-1 (ng/ml)	282 (186; 546)	220 (142; 359)	− 50.4 [− 102; − 20.9]	0.001
Urine/plasma -ratio of SDC-1	2.51 (1.57; 4.19)	1.78 (1.10; 2.87)	− 0.69 [− 1.13; − 0.20]	0.006
U-HA (ng/ml	110 (71; 128)	100 (68; 129)	− 17.87 [− 34.22; 3.99]	0.117
Urine/Plasma ratio of HA	1.01 (0.54; 2.24)	0.72 (0.23; 1.09)	− 0.30 [− 0.70; − 0.09]	0.011
U-sTM (ng/ml)	28.4 (14.5; 39.4)	18.8 (9.14; 28.5)	− 4.48 [− 9.78; − 0.49]	0.033
Urine/plasma -ratio of sTM	4.80 (2.10; 8.90)	2.60 (1.12; 5.91)	− 0.60 [− 1.63; 0.13]	0.084
U-ANG-2 (ng/ml)	0.19 (0.09; 1.22)	0.26 (0.06; 0.90)	− 0.10 (− 0.07; 0.05)	0.843
Urine/Plasma ratio of ANG-2	0.03 (0.01; 0.14)	0.03 (0.01; 0.13)	0.00 [− 0.01; 0.00]	0.366

^a^Median regression analysis, adjusted to sepsis and AKI and the baseline values of each variable. Values are Median (interquartile range) if not otherwise stated.

Three ANG-2-concentrations fell below the lower detection limit in FB-group and one in the FU-group.

ACR: albumin to creatinine ratio in urine, SDC-1: syndecan-1, HA: hyaluronan, sTM: soluble thrombomodulin, ANG-2: angiopoietin 2.

### Associations of biomarkers of endothelial dysfunction at baseline

The different components of endothelial activation had a weak to moderate, positive correlation with each other at baseline (Supplemental Table S9). The strongest correlations were seen with SDC-1 and HA (R = 0,572, *p* < 0,001, n = 128), sTM and HA (R = 0,515, *p* 0,001, n = 128), and with ANG-2 and HA (R = 0,539, *p* 0,001, n = 128).

### Association of baseline ANG-2 to changes in biomarker concentrations

Changes in the endothelial biomarker concentrations did not associate with the baseline ANG-2 concentration in either of the groups (Supplemental Table S10).

### The increase in hyaluronan in response to fluid bolus was associated with increase in Tie1

The increase in HA plasma concentration after the fluid bolus correlated moderately with the increase in Tie1 plasma concentration (R = 0,442, n = 65, *p* < 0.001) (Supplemental Table S11). No correlation between HA and Tie1 was seen in FU group (data not shown). A weak correlation existed between the increase in HA and increase in sTM plasma concentrations after the fluid bolus (R = 0,343, n = 66, *p* = 0,005).

## Discussion

We compared the immediate effect of follow-up strategy to that of a single bolus of 500 ml Ringer’s acetate on endothelial biomarkers in ICU patients who already had received marked fluid resuscitation prior study inclusion. Plasma level of the putative EG marker HA increased in the FB group independently of the baseline endothelial activation, estimated as baseline ANG-2 level, or of its renal clearance. We did not detect readily apparent differences in other endothelium-related biomarkers, but the increase in plasma level of HA correlated with the increase in plasma level of Tie1 in the FB group.

The magnitude of the rise in HA level in the FB-group shared remarkable similarity with that seen in healthy volunteers^28,29^. Associations seen between fluid-therapy and increased HA levels in animal studies also support a universal phenomenon^31,32^. Previous findings concerning critically ill patients have had some variability. Smart et al. (2017) observed that HA level associated with the volume of resuscitation fluids in patients with either sepsis or milder infection during the first 3 h of treatment independently of illness severity^33^. In line with that, restrictive fluid resuscitation has been associated with greater downward pattern of HA compared to standard fluid resuscitation during the first 6h of treatment of sepsis^34^. However, a study found no relationship between the volume of fluids given to patients with suspected sepsis and the concentration of HA, when blood was sampled at the admission and then 24h later^13^. Our findings strongly support that fluid bolus therapy increases the amount of circulating HA. There are some potential reasons for seeming inconsistencies. A possibility is that transient fluctuations in plasma levels of HA remain undetected. The half-life of HA is normally in the range of minutes^35^, its level rises rapidly when fluid therapy is started^28,29^, and may stabilize or even decrease during further treatment^34^. Also, the size of the fluctuations may vary. Although the overall effect was similar in magnitude to that seen in healthy volunteers, we cannot exclude the effect being larger in septic versus in non-septic patients as our subgroup analyses suggested. The benefit of fluid therapy may rapidly decrease after the initial resuscitation and become more harmful thereafter^36^. Yet, the existing evidence does not support that the fluid-related increase in the plasma level of HA itself depends on the timing of the therapy. Earlier studies with parallel results with ours have focused on the initial resuscitation or single experimental fluid boluses^28,29,33,34^, whereas our study intervention was introduced relatively late during the course of the acute illness.

The exact mechanism behind the fluid-related increase in HA plasma level is not known. Overall, the suggested mechanisms inducing the shedding of the EG include the activation of the shedding enzymes such as matrix metalloproteinases (MMPs) and heparanase by reactive oxygen species (ROS) and pro-inflammatory cytokines such as tumor necrosis factor α (TNF-α) or interleukin-1 β (IL-1β) as well as direct effect of ROS^17^. Factors related specifically to fluid-related shedding of the EG are increased expression of MMPs because of acute stretch in vascular wall, oscillatory shear stress and secondary activation of heparanase, activation of leucocyte mediated glycocalyx damage and possibly natriuretic peptide release^37^. Also, an ischemia–reperfusion injury with an increased burden of ROS following capillary recruitment is possible. Other possible explanations for increased plasma concentration of HA include increased hydrostatic pressure and interstitial or lymphatic flush-out^38,39^. In our study, the positive correlation between the changes in concentrations of HA and endothelium-specific Tie1, and to lesser extend the weak correlation between change in HA and sTM levels in the FB-group suggest an endothelium as a relevant source. However, ANG-Tie system is expressed also in lymphatic vessels, and thus lymphatic endothelium as a source cannot be excluded^22^. Yet, direct visualization of microcirculation has showed reduced glycocalyx thickness after comparable fluid boluses in septic and in surgical patients with similar time frame as used in our study^40^. Furthermore, several authors have shown correlation with visually detected EG disruption and plasma HA concentration^41–43^.

Contrary to previous studies assessing healthy volunteers or surgical patients^9,10,44,45^, and a study on sepsis patients^11^, we did not detect a fluid-related effect on the other EG marker, SDC-1. It is possible that ongoing fluids or previous more vigorous resuscitation already have increased the levels obscuring any additional effect. Also, the same could happen because of the decreased renal clearance in our oliguric trial patients. Small changes from the high and variable baseline levels (compared to those in healthy people) are difficult to detect, increasing the risk for type II error. In addition, a remnant of increased intravascular volume may have masked small changes in our study, as a trend toward increased SDC-1 concentration/Hb ratio was detected in the FB group. However, others have suggested that no association between fluids given, and SDC-1 level exists in acutely ill patients^33,34,46,47^. Instead, SDC-1 levels have rather indicated disease severity, need for further fluid-delivery, and prognosis^47,48^. We cannot exclude this possibility.

The clinical significance of the increase in plasma level of HA is not clear. HA affects the essential functions of EG^17^. In addition to this, it is an active signaling molecule with anti-inflammatory or pro-inflammatory effects depending on its molecular size and the surrounding molecular milieu^49^. However, considering the wide range of situations where the fluid-related increase in HA concentration has been shown, it may not be detrimental in general. A possibility is that the consequences of fluid-therapy differ between patients and within the course of care and may depend on the underlying disease pathology and host response. In this case, it would be important to be able to detect those patients with increased risk for harm. From this point of view, the modulatory role of Tie1 is interesting. Tie1 can form heterodimers with Tie2^50,51^. This heterodimer formation in certain states is crucial for the agonist function of ANG2 (contrary to antagonist function) and important in enforcing the activity of the vascular integrity promoting Tie2^19,50,51^. Both inflammation and disruption of EG can lead to decreased function of Tie1 and subsequently reduced Tie2 activation^19,50,51^. First, inflammation has been shown to lead to cleavage of the ectodomain of Tie1 necessary for the dimerization^50,51^. Second, the sulfated glycosaminoglycan heparan sulfate in the EG has been shown to form ionic interactions with Tie1—a process that helps to stabilize Tie1-Tie2 heterodimers^19^. Thus, in a state with already marked Tie1 cleavage, any additional EG loss would fortify the positive feedback mechanism for suppressed Tie2-signaling. This vicious circle would also hinder the EG repair as Tie2-signaling is necessary for the EG synthesis^20^. Vice versa, in quiescent states, as in elective surgery, fluid therapy alone may not have sufficient impact to destabilize the robust system and any EG disruption can be rapidly replaced. Not surprisingly, we did not detect immediate differences in ANG2 or Tie2 levels between treatment groups aligning with previous results from heathy adults^9^. In contrast, we found a moderate correlation with the increases in HA and Tie1 concentrations in the fluid bolus group. Assuming that HA fluctuation in plasma reflects, at least partly, the overall fluid-related effect on the EG, simultaneous Tie1 surge could possible detect those patients with underlying shift towards ANG-Tie2 suppression. Such signal could warn about increased risk for vascular leakiness, microvascular perfusion disturbances and edema formation with continued fluid administration. These hypotheses remain to be tested.

The strengths of our study include complete plasma sampling and repeated measurements. The trial was randomized, and the study groups were well balanced allowing us to dissect the fluid-related response from confounders, especially from the effect of diminished renal clearance. However, our study has some limitations. First, the study was powered according to the primary clinical endpoint of the parent RESPONSE trial and, thus, most of the results must be interpreted as hypothesis-generating only. Second, we performed ELISA-analyses for markers other than Tie1 in singlets. Third, to avoid the need for rescue fluid boluses out of the protocol the patients had to be hemodynamically relatively stable. This led to exclusion of potentially eligible patients, which reduces the generalizability. On the other hand, we did not exclude patients on the basis of their disease etiology, which increases the generalizability. Fourth, the amount of fluid given during the trial compared with the volume of fluid that patients had received beforehand may have been too small to induce a detectable change, but it was clinically reasonable and ethically acceptable. Fifth, the renal clearances are estimates for the oliguric to anuric trial period with inevitable imprecisions due to pragmatic urine sampling.

## Conclusion

A single bolus of balanced crystalloid administered beyond initial fluid resuscitation rapidly increased the plasma HA concentration independently of renal clearance. No treatment effect was detected in overt levels of other biomarkers. Whether fluid-related fluctuations in plasma biomarker levels relate to increased clinical susceptibility for fluid-related harm remains to be shown.

## Supplementary Information

Supplementary Information 1.

Supplementary Information 2.

## Data Availability

All data supporting the conclusions of this article are included within the article and its supplementary information files.

## Abbreviations

ACR: Albumin-to-creatinine ratio
AKI: Acute kidney injury
ANG-2: Angiopoietin 2
BL: Baseline
CV (%): Coefficient of variation (%)
EG: Endothelial glycocalyx
ELISA: Enzyme-linked immunosorbent assay
FB: Fluid bolus
FU: Non-interventional follow-up
HA: Hyaluronan
HS: Heparan sulfate
ICU: Intensive care unit
SDC-1: Syndecan-1
sTM: Soluble thrombomodulin
Tie1: Tyrosine-protein kinase receptor Tie-1
Tie2: Tyrosine-protein kinase receptor Tie-2
