# Supplementary material for: Fluid bolus increases plasma hyaluronan concentration compared to follow-up strategy without a bolus in oliguric intensive care unit patients
Source: Sci Rep. 2024 Sep 6;14:20808. doi: 10.1038/s41598-024-71670-2 (PMC11379687; doi:10.1038/s41598-024-71670-2)
Supplement: Supplementary file 2 — Supplementary Information 2. [file 41598_2024_71670_MOESM2_ESM.pdf]

Fluid bolus increases plasma hyaluronan concentration compared to follow-up strategy without a bolus in oliguric intensive care unit patients.

Electronic Supplementary Material (ESM)

Maija Serlo[1], Nina Inkinen[2], Päivi Lakkisto[3,4], Miia Valkonen[1], Anni Pulkkinen[2], Tuomas Selander[5], Ville Pettilä[1] and Suvi T. Vaara[1]

[1]Department of Perioperative and Intensive Care, Helsinki University Hospital, Intensive and Intermediate Care Unit, Meilahti Bridge Hospital, P.O. Box 320, 00290 HUS, Helsinki, Finland.

[2]Department of Anesthesia and Intensive Care, Central Finland Hospital Nova, Central Finland Health Care District, Hoitajantie 3, 40620, Jyväskylä, Finland.

[3]Department of Clinical Chemistry and Hematology, University of Helsinki and Helsinki University Hospital

[4] Minerva Foundation Institute for Medical Research, Helsinki, Finland

[5]Science Service Center, Kuopio University Hospital, Kuopio, Finland.

Contents

Supplementary methods 3
Enzyme-Linked Immunosorbent Assays 3
Tie1 ELISA 3
Supplemental Tables 5
Supplemental Table S1. Inclusion and exclusion criteria 5
Supplemental Table S2. Baseline characteristics of patients with missing urine samples vs patients with complete urine samples 7
Supplemental Table S3. CV-% for ELISAs 8
Supplemental Table S4. Treatment comparison with unadjusted analysis for plasma biomarkers 9
Supplemental Table S5. Treatment comparison with plasma biomarkers normalized to concomitantly measured Hb level 10
Supplemental Table S6. Treatment comparison in sepsis patients. Plasma biomarkers. 11
Supplemental Table S7. Treatment comparison in AKI-patients. Plasma biomarkers 12
Supplemental Table S8. Treatment comparison in per-protocol -population. Plasma biomarkers. 13
Supplemental Table S9. Biomarker correlations at baseline 14
Supplemental Table S10. Correlations of changes in plasma biomarkers with the baseline concentration of endothelial dysfunction marker angiopoietin 2. 15
Supplemental Table S11. Associations of the change in plasma hyaluronan concentration to changes in other plasma biomarkers in response to a fluid bolus. 16

Supplementary methods

Enzyme-Linked Immunosorbent Assays

We performed ELISAs using commercially available kits for SDC-1 (Human sCD138, cat No. 950.640.192, Diaclone SAS, Besançon, France), for HA, s-TM, ANG-2 and for Tie2 (Quantikine™ ELISA, cat No. DHYAL0, DTHBD0, DANG20 and DTE200, respectively, R&D systems, Minneapolis, USA)according to the respective protocols provided by the manufacturers. Tie1 was measured from plasma samples using sandwich ELISA developed with a reagent kits (Human Tie-1 DuoSet® ELISA development system, cat No. DY5907, R&D Systems, Minneapolis, USA) and DuoSet® Ancillary Reagent Kit 2 (cat No. DY008, R&D Systems) following the manufacturers protocol.

Other biomarkers were analyzed in singlets, except Tie1 which was analyzed in duplicates. We diluted the samples to reach the dynamic range of each assay. Measurements for Tie2 and Tie1 were applicable only for plasma, all others were measured from both the urine and from the plasma samples. Intra-assay coefficient of variation CV (%) was calculated for each assay using duplicate standards and inter-assay CV (%) using the duplicate low and high standards of each plate. Only standards spanning the typical concentration range of our specimens were used in the calculations.

Tie1 ELISA

Tie1 was measured from plasma samples using Human Tie-1 DuoSet® ELISA development system (cat No. DY5907, R&D Systems, Minneapolis, USA) and DuoSet® Ancillary Reagent Kit 2 (cat No. DY008, R&D Systems) according to manufacturer's instructions. In brief, 96-well-plates were coated with 100 µl of mouse Anti-human Tie-1 capture antibody diluted to the working concentration (4 µg/ml) in phosphate buffered saline (PBS) and incubated overnight at room temperature (RT). The wells were washed three times with 0.05%

Tween® 20 in PBS. All washing cycles were performed using Wellwash Versa Microplate Washer (ThermoScientific, Massachusetts, USA). After coating, the plates were blocked at RT using 300 µl of Reagent Diluent (1% bovine serum albumin (BSA) in PBS) for a minimum of 1hour and washed 3 x with 0.05% Tween® 20 in PBS. 100 µl of samples and standards diluted in Reagent Diluent were added to plates in duplicates and incubated for 2 hours at RT and then washed 3 x with 0.05% Tween® 20 in PBS. Up to 1:80 dilutions of samples were used when appropriate. 100 µl of Biotinylated Goat Anti-Human Tie-1 detection antibody diluted in Reagent Diluent (working concentration 200 ng/ml) was added to each well and incubated for 2 hours at RT before washing 3 x with 0.05% Tween® 20 in PBS. Streptavidin conjugated horseradish peroxidase was added for twenty minutes avoiding light exposure and then washed 3 x with 0.05% Tween® 20 in PBS. 100 µl of substrate solution (1:1 mixture of Color Reagent A (H2O2) and Color Reagent B (Tetramethylbenzidine)) was added for the next 20 minutes avoiding direct light exposure. Colorimetric reaction was stopped by adding 50 µl of Stop solution (2 N H2SO4). Optical densities were determined using a microplate reader (EnSpire 2300 Multimode Plate Reader, Perkin Elmer, Massachusetts, USA) set at 550nm and 450nm and subtracting the readings at 550nm from the readings at 450nm. Results were calculated according to manufacturer's instructions by averaging the duplicate subtracted readings and read from the standard curve generated based on a four-parameter logistic (4-PL) curve-fit using EnSpire Software (version 4.13.3005.1482, Perkin Elmer, Massachusetts, USA).

Supplemental Tables

Supplemental Table S1. Inclusion and exclusion criteria

**Inclusion criterion (all must be fulfilled)**	**Exclusion criterion (none can be present)**
18 year or older on the randomization day	Marked fluctuations in hemodynamics within the last 2 hours pre-randomization (cardiac arrhythmias affecting blood pressure, increase in norepinephrine need over 0.2 microg/kg/min, need for initiation of inotrope/inodilator)
Emergency admission to an ICU<br>• for example, admission for post operative care after elective surgery is not eligible	Administration of furosemide within last 6 hours
Oliguria (urine output < 0,5ml/kg/h) for at least two consecutive hours<br>• Patient body weight is that registered on ICU admission	Chronic kidney disease (estimated pre-critical illness GFR < 60mL/min/1.73m2)
Mean arterial pressure (MAP) >65 mmHg (with vasopressors if needed) and initial fluid resuscitation (over 20ml/kg iv-fluids during the last 12hrs) for shock/hypovolemia has been given or the patient has been in the ICU over 6hrs<br>• Iv-fluids includes crystalloids and blood products, but not carrier fluids for medication	Renal replacement therapy (RRT)<br>• RRT has been already started in the ICU for AKI<br>• Commencing RRT (according to last laboratory values) is likely within the next 6hrs<br>• Patient undergoes regular (chronic) dialyses<br>• Patient has a history of kidney transplantation
	Urgent indications for commencing RRT for AKI are present (based on last blood work)<br>• plasma potassium > 6mmol/L<br>• severe metabolic acidosis (pH<7.20 and bicarbonate <12mmol/L),<br>• evidence of severe respiratory failure (PaO2/FiO2 ratio <200) and clinical perception of volume overload<br>• AKI has continued over 72hrs (creatinine remains more than twice the normal level/oliguria continues)
	Fluid overload (cumulative fluid accumulation exceeds 10% of baseline body weight)<br>• Even if fluid overload has no impact on

	oxygenation
	Pulmonary edema (bilateral infiltrates in chest x-ray)
	Active bleeding (need for transfusion, platelets, or fresh frozen plasma)<br>• operational definition: transfusion is planned within next 6 hrs
	Suspected or known intra-abdominal hypertension (intra-abdominal pressure >16mmHg)
	Pregnant or lactating<br>• clinical team being aware of pregnancy/lactation
	Expected survival less than 24h<br>• Patients whose ICU treatment is withdrawn<br>• Strong suspicion that patient will not survive over 24hrs<br>• Organ donors
	Obtaining informed written consent is not possible / consent is denied

Supplemental Table S2. Baseline characteristics of patients with missing urine samples vs patients with complete urine samples.

Characteristic	Urine sample(s) missing (n=22)	Urine sample(s) not missing (n=108)	P
Age (years)	68 (59-76)	69 (56-74))	0.859
Sex; male (%)	14 (63.6)	71 (65.7)	0.850
Weight (kg)	82 (70-90)	87 (87 -102)	0.080
SAPS II score	46 (34 -56)	39 (31-45)	0.039
Surgical admission n (%)	12 (54.5)	58 (53.7)	0.942
Time from the admission to ICU, h	21.2 (12.3 – 26.4)	19.4 (11.3 -35.5)	0.497
AKI, (%)	14 (63.6)	63 (58.3)	0.645
Sepsis, (%)	15 (68.2)	57 (52.8)	0.185
Cumulative balance of fluids at ICU before randomization (ml)	1902 (1656-25540)	1962 (1198-3755)	0.938
Previous 24h intake of fluids (ml)	3610 (2762; 6030)	4209 (2920; 5454)	0.630
SOFA score	9 (7-11)	7 (5-9)	0.002
Plasma biomarkers for glycocalyx disruption (ng/ml)			
SDC-1	190 (89.7-379)	121 (74.7-252.7)	0.131
HA	123.5 (53.2-2219.1)	112.2 (50.1-259.4)	0.249
Plasma biomarker of endothelial cell injury (ng/ml)			
sTM	9.37 (5.05-13.3)	6.10 (4.03-10.09)	0.029
Plasma biomarkers for endothelial dysregulation/activation (ng/ml)			
ANG-2	8.58 (4.54-40.56)	7.18 (4.53-12.93)	0.193
Tie2	15.9 (11.01-19.78)	14.7 (11.43-19.74)	0.975
Tie1	30.7 (18.0-61.7)	21.1 (15.7-34.4)	0.047

Mann-Whitney U Continuous data compared with Mann-Whitney U, proportions with Pearson Chi-square. Abbreviations: SDC-1: syndecan-1, HA: hyaluronan, sTM: soluble thrombomodulin, ANG-2: angiopoietin 2, tie2 and tie1: transmembrane tyrosine kinase receptor 2 and 1.

Supplemental Table S3. CV-% for ELISAs

Assay	Intra-assay CV (%)	Inter-assay CV (%)
SDC-1	9.3	11.8
HA	9.9	5.4
sTM	1.4	3.4
ANG-2	2.8	7.7
Tie2	6.4	9.1
Tie1	4.8	7.7

Supplemental Table S4. Treatment comparison with unadjusted analysis for plasma biomarkers.

**Biomarker concentration (ng/ml)**		*Baseline* (pre)	*After the study period* (post)	Median regression analysis<br>*Difference in medians (95% CI)*	*P**	*n*
**SDC-1**						
	**FU**	133.01 (87.52; 264.89)	133.79 (80.17; 280.28)			63
	**FB**	138.21 (71.36; 258.56)	140.21 (81.05; 256.79)	4.53 (-4.81; 19.3)	0.212	67
**HA**						
	**FU**	117 (54.0; 260)	119 (43.0; 324)			62
	**FB**	112 (49.4; 327)	156 (69.0; 418)	32.1 (16.2; 55.5)	<0.001	66
**sTM**						
	**FU**	6.50 (4.04; 9.95)	5.98 (4.39; 9.57)			63
	**FB**	6.12 (4.36; 11.57)	7.00 (4.23; 10.46)	-0.35 (-0.86; 0.39)	0.345	67
**ANG-2**						
	**FU**	7.40 (4.67; 12.70)	7.47 (4.54; 12.61)			63
	**FB**	7.18 (4.42; 14.05)	7.44 (4.32; 13.81)	0.03 (-0.52; 0.64)	0.992	67
**Tie2**						
	**FU**	14.97 (11.19; 19.81)	15.03 (12.44; 20.25)			63
	**FB**	14.84 (11.25; 19.51)	14.09 (10.92; 19.48)	0.37 (-1.36; 1.74)	0.717	67
**Tie1**						
	**FU**	21.42 (16.82; 35.12)	23.42 (18.28; 32.60)			62
	**FB**	21.31 (15.35; 39.49)	23.06 (16.86; 35.46)	-0.23 (-3.27; 2.66)	0.999	66

*Median regression analysis. Adjusted to baseline values only, not to stratification factors “sepsis” or “AKI”.

Pre and post data are presented as medians and interquartile range. Medians and quartiles are calculated without exclusions (vs listwise exclusion in the regression analysis if appropriate) and without adjustments.

Supplemental Table S5. Treatment comparison with plasma biomarkers normalized to concomitantly measured Hb level.

**Biomarker concentration (ng/mg Hb)**		*Baseline* (pre)	*After the study period* (post)	Median regression analysis: *Difference in medians (95% CI)*	*P**	*n*
**SDC-1**						
	**FU**	1.21 (0.73; 2.11)	1.23 (0.68; 2.10)			59
	**FB**	1.20 (0.63; 2.61)	1.34 (0.81; 2.50)	0.1 (-0.00; 0.24)	0.051	64
**HA**						
	**FU**	1.00 (0.50; 2.41)	1.04 (0.437; 3.02)			58
	**FB**	1.01 (0.42; 2.67)	1.38 (0.631; 3.91)	0.32 (0.15; 0.62)	<0.001	62
**sTM**						
	**FU**	0.0569 (0.0337; 0.0887)	0.0516 (0.0380; 0.0849)			59
	**FB**	0.0633 (0.0401; -0.1076)	0.0684 (0.0392; 0.1038)	0.00 (-0.01; 0.01)	0.344	64
**ANG-2**						
	**FU**	0.0659 (0.0410; 0.1112)	0.0715 (0.0412; 0.1216)			59
	**FB**	0.0700 (0.0416; 0.1243)	0.0732 (0.0417; 0.1351)	0.00 (-0.01; 0.01)	0.109	64
**Tie2**						
	**FU**	0.128 (0.097; 0.177)	0.135 (0.107; 0.167)			59
	**FB**	0.129 (0.101; 0.195)	0.140 (0.107; 0.200)	0.01 (-0.01; 0.02)	0.496	54
**Tie1**						
	**FU**	0.201 (0.140; 0.291)	0.224 (0.150; 0.319)			58
	**FB**	0.203 (0.142; 0.361)	0.224 (0.151; 0.325)	0.00 (-0.03; 0.03)	0.954	63

*Median regression analysis, adjusted for AKI, sepsis and baseline concentration of each biomarker.

Pre and post data are presented as medians and interquartile range. Medians and quartiles are calculated without exclusions (vs listwise exclusion in the regression analysis if appropriate) and without adjustments.

Supplemental Table S6. Treatment comparison in sepsis patients. Plasma biomarkers.

Biomarker concentration ng/ml		*Baseline* (pre)	*After the study period* (post)	Median regression analysis		
				Difference in medians (95% CI)	*P**	*n*
**SDC**						
	**FU**	168.23 (110.70; 303.93)	167.43 (128.88; 365.27)		0.885	35
	**FB**	168.23 (95.68; 305.31)	151.84 (100.34; 276.29)	2.40 (-24.0; 29.0)		37
**HA**						
	**FU**	176 (88.0; 637)	144 (92.2; 431)		0.001	34
	**FB**	205 (69.2; 436)	255 (94.2; 541)	56.9 (28.9; 109)		37
**sTM**						
	**FU**	8.472 (5.059; 11.90)	8.366 (5.625; 11.83)		0.788	35
	**FB**	6.487 (5.037; 12.31)	7.376 (4.572; 10.90)	-0.20 (-1.06; 0.85)		37
**ANG-2**						
	**FU**	8.917 (6.304; 16.10)	8.738 (6.153; 15.48)		0.400	35
	**FB**	8.528 (5.365; 17.16)	8.569 (6.162; 16.62)	0.6 (-0.59; 1.07)		37
**Tie2**						
	**FU**	17.87 (13.83; 20.60)	16.36 (13.79; 22.10)		0.234	35
	**FB**	17.83 (11.55; 21.35)	17.02 (12.51; 21.49)	1.20 (-0.92; 3.51)		37
**Tie1**						
	**FU**	20.90 (17.68; 35.16)	23.49 (19.50; 34.00)		0.895	34
	**FB**	27.04 (15.17; 43.76)	24.59 (16.47; 36.89)	-0.90 (-5.56; 3.73)		36

*Median regression analysis, adjusted for AKI and baseline concentration of each biomarker.

Pre and post data are presented as medians and interquartile range. Medians and quartiles are calculated without exclusions (vs listwise exclusion in the regression analysis if appropriate) and without adjustments.

Supplemental Table S7. Treatment comparison in AKI-patients. Plasma biomarkers.

Biomarker concentration ng/ml		*Baseline* (pre)	*After the study period* (post)	Median regression analysis		
				Difference in medians (95% CI)	*P**	*n*
**SDC**						
	**FU**	142.60 (98.92; 278.22)	146.63 (99.92; 298.31)			37
	**FB**	159.02 (83.98; 315.32)	162.63 (94.58; 292.53)	2.60 (-15.4; 23.7)	0.626	40
**HA**						
	**FU**	128 (55.3; 309)	132 (46.6; 358)			36
	**FB**	131 (55.4; 329)	176 (61.5; 411)	20.5 (4.29; 45.5)	0.009	40
**sTM**						
	**FU**	6.968 (4.850; 10.52)	6.327 (5.027; 10.12)			37
	**FB**	7.597 (4.997; 12.29)	7.658 (4.439; 12.42)	-0.30 (-1.27; 0.64)	0.559	40
**ANG-2**						
	**FU**	8.974 (6.410; 17.47)	10.55 (6.798; 16.84)			37
	**FB**	8.486 (5.601; 16.40)	7.957 (6.606; 17.51)	-0.20 (-0.82; 1.23)	0.883	40
**Tie2**						
	**FU**	15.08 (11.41; 19.70)	15.34 (13.12; 18.34)			37
	**FB**	16.21 (11.68; 20.79)	16.30 (11.88; 19.60)	-0.60 (-2.24; 2.17)	0.893	40
**Tie1**						
	**FU**	21.46 (18.35; 33.29)	27.52 (21.95; 34.04)			36
	**FB**	28.08 (19.01; 46.17)	26.63 (17.98; 37.62)	-2.50 (-7.67; 1.03)	0.176	39

*Median regression analysis, adjusted for sepsis and baseline concentration of each biomarker.

Pre and post data are presented as medians and interquartile range. Medians and quartiles are calculated without exclusions (vs listwise exclusion in the regression analysis if appropriate) and without adjustments.

Supplemental Table S8. Treatment comparison in per-protocol -population. Plasma biomarkers.

Biomarker concentration ng/ml		Baseline (pre)	After the study period (post)	Median regression analysis: Difference in medians (95% CI)	P*	n
**SDC**						
	**FU**	130.3 (87.93; 257.7)	133.6 (80.63; 238.1)			60
	**FB**	143.6 (70.86; 262.3)	149.2 (70.24; 257.1)	7.10 (-6.90; 19.9)	0.374	62
**HA**						
	**FU**	121 (55.2; 259)	122 (53.5; 313)			59
	**FB**	109 (45.3; 274)	136 (62.7; 350)	25.7 (12.7; 53.4)	0.001	62
**sTM**						
	**FU**	6.542 (4.016; 10.07)	6.106 (4.412; 9.483)			60
	**FB**	6.098 (4.060; 11.54)	6.448 (3.924; 10.45)	-0.14 (-0.90; 0.45)	0.566	62
**ANG-2**						
	**FU**	7.628 (4.843; 13.46)	7.515 (4.987; 12.71)			60
	**FB**	7.271 (4.412; 14.05)	7.449 (4.299; 13.84)	0.11 (-0.36; 0.72)	0.670	62
**Tie2**						
	**FU**	14.85 (11.12; 19.44)	15.00 (12.48; 19.41)			60
	**FB**	14.78 (11.22; 19.44)	14.04 (11.47; 18.81)	0.24 (-1.87; 1.89)	0.866	62
**Tie1**						
	**FU**	20.90 (17.68; 35.16)	23.49 (19.50; 34.00)			34
	**FB**	27.04 (15.17; 43.76)	24.59 (16.47; 36.89)	-0.90 (-5.56; 3.73)	0.895	36

*Median regression analysis, adjusted for AKI, sepsis and baseline concentration of each biomarker.

Pre and post data are presented as medians and interquartile range. Medians and quartiles are calculated without exclusions (vs listwise exclusion in the regression analysis if appropriate) and without adjustments.

Supplemental Table S9. Biomarker correlations at baseline.

		SDC-1	HA	sTM	ANG-2	Tie2
SDC-1	Spearman's rho r	-				
	p (two-tailed)					
	n					
HA	Spearman's rho r	.572	-			
	p (two-tailed)	<0.001				
	n	128				
sTM	Spearman's rho r	.391	.515	-		
	p (two-tailed)	<0.001	<0.001			
	n	130	128			
ANG-2	Spearman's rho r	.452	.539	.307	-	
	p (two-tailed)	<0.001	<0.001	<0.001		
	n	130	128	130		
Tie2	Spearman's rho r	.282	.330	.219	.254	-
	p (two-tailed)	0.001	<0.001	0.012	0.004	
	n	130	128	130	130	
Tie1	Spearman's rho r	.233	.292	.208	.367	.246
	p (two-tailed)	0.008	<0.001	0.018	<0.001	0.005
	n	129	127	129	129	129

Supplemental Table S10. Correlations of changes in plasma biomarkers with the baseline concentration of endothelial dysfunction marker angiopoietin 2.

Change in biomarker concentration (post-pre)		Spearman's rho r	*p* (two-tailed)	*n*
SDC-1				
	FU	0.030	0.815	63
	FB	-0.015	0.903	67
HA				
	FU	0.025	0.847	61
	FB	0.100	0.426	66
sTM				
	FU	0.035	0.786	63
	FB	-0.082	0.509	67
ANG-2				
	FU	-0.238	0.061	63
	FB	-0.142	0.251	67
Tie2				
	FU	-0.171	0.181	63
	FB	0.133	0.283	67
Tie1				
	FU	0.188	0.143	62
	FB	-0.168	0.177	66

Supplemental Table S11. Associations of the change in plasma hyaluronan concentration to changes in other plasma biomarkers in response to a fluid bolus.

Delta biomarker concentration (post-pre)	Spearman's rho r	*p* (two-tailed)	95% CI	*n*
SDC-1	0.023	0.853	-0.288; 0.376	66
sTM	0.343	0.005	-0.056; 0.563	66
ANG-2	0.104	0.405	-0.341; 0.324	66
Tie2	0.082	0.511	-0.162; 0.484	66
Tie1	0.442	<0.001	0.003; 0.608	65
